# Phosphorolytic degradation of leaf starch via plastidic α-glucan phosphorylase leads to optimized plant growth and water use efficiency over the diel phases of Crassulacean acid metabolism

**DOI:** 10.1093/jxb/erab132

**Published:** 2021-03-22

**Authors:** Nathalie Ceusters, Johan Ceusters, Natalia Hurtado-Castano, Louisa V Dever, Susanna F Boxall, Jana Kneřová, Jade L Waller, Rebecca Rodick, Wim Van den Ende, James Hartwell, Anne M Borland

**Affiliations:** 1 Faculty of Engineering Technology, Department of Biosystems, Division of Crop Biotechnics, Campus Geel, KU Leuven, Kleinhoefstraat 4, 2440 Geel, Belgium; 2 UHasselt, Centre for Environmental Sciences, Environmental Biology, Campus Diepenbeek, Agoralaan Building D, 3590 Diepenbeek, Belgium; 3 School of Natural and Environmental Sciences, Newcastle University, Newcastle upon Tyne, UK; 4 Department of Molecular Biology and Biotechnology, University of Sheffield, Sheffield, UK; 5 Department of Biochemistry and Systems Biology, Institute of Systems, Molecular and Integrative Biology, University of Liverpool, Liverpool, UK; 6 Faculty of Science, Department of Biology, Laboratory of Molecular Plant Biology, KU Leuven, Kasteelpark Arenberg 31, B-3001 Heverlee, Belgium; 7 University of California, Davis, USA

**Keywords:** CAM, gas exchange, hydrolytic pathway, phosphorolytic pathway, starch

## Abstract

In plants with Crassulacean acid metabolism (CAM), it has been proposed that the requirement for nocturnal provision of phosphoenolpyruvate as a substrate for CO_2_ uptake has resulted in a re-routing of chloroplastic starch degradation from the amylolytic route to the phosphorolytic route. To test this hypothesis, we generated and characterized four independent RNAi lines of the obligate CAM species *Kalanchoë fedtschenkoi* with a >10-fold reduction in transcript abundance of plastidic α-glucan phosphorylase (PHS1). The *rPHS1* lines showed diminished nocturnal starch degradation, reduced dark CO_2_ uptake, a reduction in diel water use efficiency (WUE), and an overall reduction in growth. A re-routing of starch degradation via the hydrolytic/amylolytic pathway was indicated by hyperaccumulation of maltose in all *rPHS1* lines. Further examination indicated that whilst operation of the core circadian clock was not compromised, plasticity in modulating net dark CO_2_ uptake in response to changing photoperiods was curtailed. The data show that phosphorolytic starch degradation is critical for efficient operation of the CAM cycle and for optimizing WUE. This finding has clear relevance for ongoing efforts to engineer CAM into non-CAM species as a means of boosting crop WUE for a warmer, drier future.

## Teaser

Characterization of RNAi lines of *Kalanchoë fedtschenkoi* deficient in chloroplastic α-glucan phosphorylase demonstrates that the phosphorolytic route of starch degradation is of central importance for CAM in this model species.

## Introduction

In plants with Crassulacean acid metabolism (CAM), the nocturnal production of phosphosphoenolpyruvate (PEP) is a key limiting factor for night-time CO_2_ uptake (Dodd *et al.*, 2003; Ceusters *et al.*, 2008, 2010). In starch-storing CAM plants such as the model species *Kalanchoë fedtschenkoi*, chloroplastic starch is degraded in the dark to provide PEP as substrate for PEP carboxylase (PEPC), the enzyme responsible for CO_2_ uptake at night (Hartwell *et al.*, 2016; Yang *et al.*, 2017). In nocturnal Phase I, PEPC combines PEP with atmospheric and/or respiratory CO_2_ (as HCO_3_^–^) to form oxaloacetate, which is then converted to malic acid and stored in the vacuole. In Phase II, a transient surge of net CO_2_ uptake may occur at the start of the photoperiod. Phase III commences as malate exits the vacuole and decarboxylation releases CO_2_, which is re-fixed by Rubisco and processed to sucrose and/or starch via the photosynthetic carbon reduction cycle. During this daytime phase of CAM, the CO_2_ concentration inside the leaf rises in the light due to malate decarboxylation, stomata close, and transpirational water loss is curtailed. Later in the photoperiod, when malate decarboxylation is complete, stomata may re-open and net CO_2_ uptake occurs (Phase IV of CAM). Nocturnal PEP production represents a significant sink for carbohydrate that must be balanced alongside the supply of carbohydrates to fuel growth and maintenance (Borland *et al.*, 2016). Deeper understanding of the mechanisms that CAM plants use to optimize net carbon gain and water use efficiency (WUE) requires identification and functional characterization of the enzymes and transporters responsible for nocturnal starch degradation in plants with this photosynthetic specialization. Aspirations to engineer CAM into non-CAM species as a means of improving plant WUE (Borland *et al.*, 2014) also demand better understanding of starch degradation in CAM to establish whether the nocturnal generation of PEP will require a re-routing of starch degradation in C_3_ plants (Borland *et al.*, 2016).

**Fig. 1.**
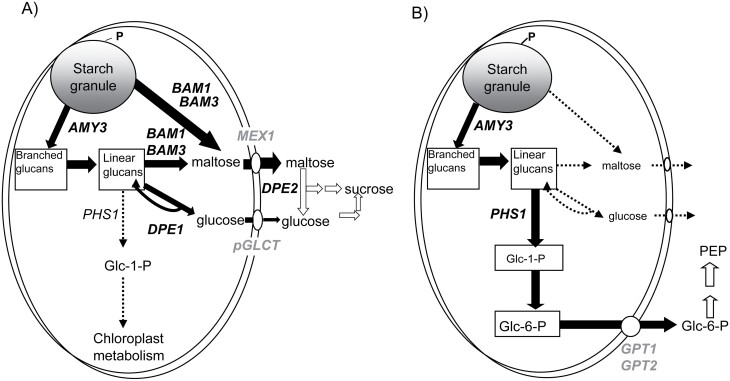
Summary outline of metabolic steps implicated in nocturnal degradation of leaf starch: (A) in *Arabidopsis thaliana* (C_3_) for the provision of sucrose and (B) in CAM for the provision of PEP (phosphoenolpyruvate). Enzymes shown include: AMY3 (α-amylase 3); BAM1 (β-amylase 1); BAM3 (β-amylase 3); DPE1 (chloroplastic disproportionating enzyme); PHS1 (chloroplastic α-glucan phosphorylase); and cytosolic disproportionating enzyme (DPE2). Transporters are shown in grey text and include: MEX1 (maltose transporter); pGLCT (plastidic glucose transporter), GPT1 (glucose phosphate translocator 1); and GPT2 (glucose phosphate translocator 2).

Multiple enzymes and transporters have been implicated in the degradation of leaf starch and operate via intricate networks subject to circadian and metabolic control (Weise *et al.*, 2006, 2011). In the C_3_ plant Arabidopsis, β-amylases (BAMs; mainly plastidic BAM3) are the principle amylolytic enzymes responsible for hydrolytic starch degradation at night (Smith *et al.*, 2005; Fig. 1A). BAM3 works synergistically with debranching enzymes (Zeeman *et al.*, 2007; Streb and Zeeman, 2012), which are responsible for the hydrolysis of the α-1,6-branches of starch into short soluble malto-oligosaccharides, whilst α-amylases and chloroplastic DISPROPORTIONATING ENZYME (DPE1) also play roles in the liberation and metabolism of malto-oligosaccharides in the stroma (Stitt and Zeeman, 2012; Streb and Zeeman, 2012). Maltose and glucose are the major products of starch degradation in Arabidopsis and are exported from the chloroplast by dedicated transporters, namely MALTOSE EXCESS PROTEIN1 (MEX1) and PLASTIDIC GLUCOSE TRANSPORTER (pGlcT) (Niittylä *et al.*, 2004; Smith *et al.*, 2005; Cho *et al.*, 2011). In contrast, CAM species appear to export different breakdown products from the chloroplast following starch degradation. In the starch-storing, facultative CAM species *Mesembryanthemum crystallinum* (ice plant), it was reported that chloroplasts isolated from C_3_ leaves exported mainly maltose, whilst chloroplasts isolated from CAM-induced leaves exported predominantly glucose 6-phosphate (G6P) (Neuhaus and Schulte, 1996; Kore-eda and Kanai, 1997). Subsequently, it was shown that CAM induction in *M. crystallinum* is accompanied by a substantial increase in chloroplastic G6P transport rates and a >70-fold increase in transcript abundance of the plastidic GLUCOSE 6-PHOSPHATE:PHOSPHATE TRANSLOCATOR (GPT) (Häusler *et al.*, 2000; Kore-Eda *et al.*, 2013).

Production and export of G6P from the chloroplast implies a critical role for chloroplastic α -glucan phosphorylase (PHS1) in starch degradation in CAM plants (Fig. 1B). In *Arabidopsis thaliana*, PHS1 is believed to predominantly provide substrate for internal chloroplast metabolism (Zeeman *et al.*, 2004; Weise *et al.*, 2006, 2011). PHS1 releases glucose 1-phosphate (G1P) from the non-reducing ends of the glucan chains, which, following conversion of G1P to G6P by phosphoglucomutase, results in the export of G6P from the chloroplast. This process of phosphorolytic starch degradation offers energetic advantages compared with hydrolytic degradation of starch to maltose and glucose. Glycolytic conversion of G6P to PEP in the cytosol provides ATP by substrate-level phosphorylation, and recent modelling using a flux-balance approach suggests that this would help to offset the energetic costs of the CAM cycle (Shameer *et al.*, 2018). Establishing if CAM has diverged from the C_3_ pathway in terms of the route used for starch degradation requires a targeted genetic approach to knock down the activity of PHS1. Such an approach should also reveal whether there is sufficient plasticity across the network of starch degradation to enable provision of PEP for CAM using the hydrolytic route.

In the present work, stable transgenic RNAi lines of *K. fedtschenkoi* were generated in which chloroplast-localized *KfPHS1* transcripts were down-regulated. Four independent *rPHS1* RNAi lines with a >10-fold reduction in levels of *KfPHS1* transcript were characterized in terms of growth, diel starch turnover, nocturnal acid accumulation, and patterns of 24 h light/dark leaf gas exchange. Soluble sugar profiles were examined as indicators of potential re-routing of starch degradation in response to curtailing the phosphorolytic route. The impact of loss of *KfPHS1* on the temporal coordination of the four phases of CAM-associated CO_2_ fixation and the free-running circadian rhythm of CO_2_ fixation were also characterized in detail.

## Materials and methods

### Plant materials and sampling

*Kalanchoë fedtschenkoi* Hamet et Perrier plants were propagated clonally from leaf margin adventitious plantlets using the same clonal stock originally obtained from the Royal Botanic Gardens, Kew (Wilkins, 1959). Plants were grown in 1.63 litre pots using a mixture of John Innes no. 3 compost containing one-third Perlite plus Osmocote slow-release fertilizer. Plants were grown for 8–10 weeks before sampling in controlled growth rooms with a photoperiod of 12 h, day/night temperatures of 25/19 °C, and photosynthetic photon flux density (PPFD) at plant height of ~250 µmol photons m^–2^ s^–1^ that was provided by LED growth lamps with broad wavelength spectra (Phytolux Attis: https://phytolux.com). Plants were watered from below on alternate days and maintained under ambient concentrations of CO_2_. Leaf pair 6 (LP6; numbered from leaf pair 1 flanking the shoot apical meristem) were used for gas exchange measurements, biochemical assays, and analyses of gene transcript abundance using reverse transcription–quantitative real-time PCR (RT–qPCR).

### Analysis of *PHS* nucleotide and amino acid sequences from *K. fedtschenkoi*

Predicted amino acid sequences of the previously characterized *A. thaliana* chloroplast-targeted *PHS1* (AT3G29320) and cytosolic *PHS2* (AT3G46970) (Zeeman *et al.*, 2004) were retrieved from the Arabidopsis genome database (www.arabidopsis.org). These were used in BLASTP searches with the ‘Custom BLAST’ feature of Geneious R11 (www.geneious.com) to query an in-house, chromosome-scale Hi-C scaffolded, PacBio-only *de novo* assembly of the complete genome sequence of the Kew–Glasgow–Liverpool accession of *K. fedtschenkoi* (Wilkins, 1959) and used here for subsequent transformation with the *KfPHS1* RNAi construct. The orthologous *PHS* gene sequences were also identified through BLAST searches of the published *K. fedtschenkoi* genome available for the ‘M2’ accession/ecotype via the Phytozome database (Yang *et al.*, 2017). Sequences of three *PHS* genes from both *K. fedtschenkoi* genomes were retrieved as both nucleotide and predicted amino acid sequences and used for the pairwise and multiple sequence alignments presented in Supplementary Figs S1–S3. Both the pairwise nucleotide and multiple amino acid sequence alignments were generated using the ‘Geneious Alignment’ option within Geneious R11. The phylogenetic tree was generated from the multiple amino acid sequence alignment using the in-built tree-building options in Geneious. Specifically, a Neighbor–Joining tree was generated using the Jukes–Cantor genetic distance model, and 1000 bootstraps were utilized to generate the consensus tree shown. To predict subcellular localization and transit peptide identity, the online chloroplast transit peptide and cleavage site prediction software ChloroP was used (Emanuelson *et al.*, 1999).

### Generation of transgenic *Kalanchoë fedtschenkoi* lines

Intron-containing hairpin RNAi constructs were designed to target silencing of the gene encoding α-glucan phosphorylase (EC 2.4.1.1) (*KfPHS1* KfGene000193/Kaladp0024s0136.1, *A. thaliana* orthologue *PHS1* AT3G29320). A short *KfPHS1* fragment (357 bp), spanning 47 bp of the 3′ end of the ORF including the stop codon and 310 bp of the 3′-untranslated region (UTR), was amplified from CAM leaf cDNA using high fidelity PCR with KOD Hot Start DNA Polymerase (Merck, Germany). The following primers were used: *KfPHS1_RNAi_F* CACCTCATGGGATCCCTGGAAGGA and *KfPHS1_RNAi_R* GCAGCACCAGATCAAGCATGATG. PCR products were cloned into the pENTR/D Gateway-compatible entry vector via directional TOPO cloning (Life Technologies, UK), and recombined into the intron-containing hairpin RNAi binary vector pK7GWIWG2(II) (Karimi *et al.*, 2002) using LR Clonase II enzyme mix (Life Technologies, UK). Constructs were confirmed by DNA sequencing and introduced into *Agrobacterium tumefaciens* strain GV3101 using the freeze–thaw method (Höfgen and Willmitzer, 1988). *Agrobacterium*-mediated stable transformation of *K. fedtschenkoi* was carried out using a tissue culture-based procedure described previously (Dever *et al.*, 2015). Once roots had developed, the transgenic plants were transferred to soil and grown as described above.

Bulking up of transformants occurred by clonal propagation via leaf plantlets produced around the margins of detached leaves (Garcês *et al.*, 2007; Dever *et al.*, 2015).

### High-throughput screens of leaf acidity and starch content

Leaf acidity, which served as a proxy for leaf malate content, and leaf starch content were determined using leaf discs (0.5 cm diameter) taken from leaves (LP6) sampled at the start and end of the 12 h photoperiod from both the wild type and *rPHS1* RNAi lines. The discs were stained with chlorophenol red (for acidity) and iodine solution (for starch) as described previously (Cushman *et al.*, 2008; Dever *et al.*, 2015). For each transgenic line, leaf discs were sampled in triplicate and stained in a 96-well plate format.

### RNA isolation and RT–qPCR

Four independent RNAi lines, selected on the basis of altered leaf acidity and starch content as determined above, were checked for down-regulation of endogenous *KfPHS1* transcript abundance. LP6 was sampled 2 h before the end of the 12 h dark period and at 2 h before the end of the 12 h photoperiod. Total RNA was isolated from 100 mg of frozen, ground leaf tissue using the Qiagen RNeasy kit (Qiagen, Germany) following the manufacturer’s protocol, with the addition of 14 μl of 2-mercaptoethanol and 260 μl of 50 mg ml^–1^ polyethyleneglycol (PEG) 20000 to the 430 μl of RLC buffer used for each extraction. cDNA was synthesized from the total RNA according to the manufacturer’s instructions (Qiagen, Germany). Three technical replicates and three biological replicates were analysed using the SensiFAST SYBR No Rox kit (Bioline) in an Agilent MX3005P qPCR system cycler and primers designed with Geneious version R11. Primer specificity was checked using BLAST searches, and primer efficiency was checked using a melting curve profile standard curve. In every PCR plate, an interplate calibrator (made from a pool of cDNAs assembled from *K. fedtschenkoi* wild-type LP6 samples taken every 4 h over a 12 h light/12 h dark cycle) was amplified in triplicate in order to correct any plate to plate variation. In addition, non-template controls were included in every plate to confirm the absence of contamination. Each biological replicate was assayed in triplicate, and the relative abundance of each gene was determined using Agilent MxPRO QPCR software following the manufacturer’s instructions (Agilent Technologies). Results were normalized to a *K. fedtschenkoi* orthologue of a thioesterase/thiol ester dehydrase-isomerase (TEDI) superfamily protein as used previously as the reference gene for RT–qPCR in this species (Boxall *et al.*, 2017, 2020; KfGene008730/Phytozome Kaladp0068s0118.1; Arabidopsis orthologue AT2G30720.1). RT–qPCR was also used to check diel changes in the transcript abundance of a selection of other genes encoding transporters and degradative enzymes implicated in starch degradation. All primers used for RT–qPCR analyses are listed in Supplementary Table S1.

### Growth analyses

Plants were sampled after 10 weeks of growth. Image J was used to calculate total leaf area after scanning all detached leaves. Harvested leaves plus stems were dried to constant weight in an oven at 80 °C which took 6 d, and roots were washed free of soil and perlite before drying to constant weight. Data are presented as the mean of five biological replicates ±SEM.

### Gas exchange measurements

Gas exchange parameters (net CO_2_ uptake, stomatal conductance, and transpiration) were measured for LP6 in controlled-environment chambers which provided conditions similar to those experienced during propagation. Leaves were placed into the leaf chamber of the LI-6400XT (LI-COR Inc., Lincoln, NE, USA) in the middle of the photoperiod when stomata are expected to be closed. Flow rate through the cuvette was 400 µmol s^–1^ and CO_2_ concentration was set at 400 µmol m^–1^. Block temperature was set to match that of the growth chamber during the day (25 °C), and subsequently at night (19 °C), while the cuvette light intensity tracked the diel changes in light/dark set in the growth chamber (i.e 250 µmol photons m^–2^ s^–1^ during the photoperiod). Measurements were recorded every 15 min. Each 24 h gas exchange curve presented is representative of data obtained from three independent biological replicates.

For the photoperiod shift experiments, net CO_2_ exchange of LP6 was monitored over 12 h light/12 h dark cycles using a Walz CMS-400 compact mini-cuvette system equipped with a BINOS-100 infra-red gas analyser (H. Walz GmbH, Effeltrich, Germany). The leaf was placed into a well-stirred gas exchange cuvette that tracked PPFD and temperature conditions within the plant growth chamber. Data logging occurred at 15 min intervals, and gas exchange parameters were calculated using the DIAGAS software package (H. Walz GmbH). Each gas exchange curve presented was representative of at least three independent runs on different plants.

Gas exchange measurements were also made for entire shoots comprising seven leaf pairs. Intact shoots were placed inside a Walz mini-cuvette (internal volume 460 cm^3^) which was programmed to track the diel changes in PPFD and temperature (light/dark 25/19 °C) experienced within the growth chamber. Data were logged at 15 min intervals for two complete 12 h light/12 h dark cycles. Subsequently, conditions in the growth chamber were re-set to constant light (200 µmol photons m^–2^ s^–1^ at plant height) and constant temperature (19 °C) (LL), and the cuvette was set to track these conditions for a further 95 h in order to monitor free-running circadian rhythms of net CO_2_ exchange. Representative gas exchange traces from three independent biological replicates (i.e. three separate plants) of the wild type and the *rPHS1* line are shown.

### Biochemical analyses of metabolites

LP6 was detached from the wild type and *rPHS1* lines at the end of the night and end of the photoperiod, snap-frozen in liquid N_2_, and stored at –80 °C. Metabolite extraction was conducted by heating frozen, powdered leaf tissue in 80% methanol to 70 °C for 40 min. Cooled aliquots were used for titratable acidity and soluble sugar measurements, and insoluble material was used for starch determination. Titratable acidity was determined by titration against 0.01 N NaOH to a neutral endpoint, as indicated by phenolphthalein. Starch content was determined following digestion by amyloglucosidase and α-amylase as described by Haider *et al.* (2012) and assayed for glucose equivalents using the phenol–sulfuric acid colorimetric method (Dubois *et al.*, 1956). Soluble sugars were analysed via high-pressure ion chromatography (HPIC) as described previously (Haider *et al.* 2012).

### Enzyme activities

All assays were conducted on LP6 leaves sampled towards the end of the photoperiod. Extraction and assay of PEPC were performed as described previously (Borland and Griffiths, 1997). Extraction and assay of starch phosphorylase (α-glucan phosphorylase) were also as described previously (Kumar and Sanwal, 1982). Additional starch enzyme assays [cytosolic disproportionating enzyme, BAM, maltase (α-glucosidase), and plastidic D-enzyme (4-α-glucanotransferase)] were adapted from previously published methods (Zeeman *et al.*, 1998; Chia *et al.*, 2004).

### Native PAGE

Leaves were harvested at 6 h into the 12 h photoperiod and soluble proteins were extracted as described by Taybi *et al.* (2017). Native discontinuous PAGE for starch phosphorylase isoforms was performed in separating gels containing oyster glycogen, as described previously (Steup, 1990; Zeeman *et al.*, 1998). Native gels containing amylopectin were used to indicate the complement of other starch-degrading enzymes present in leaves of the wild type and *rPHS1*. The products of hydrolysis of amylopectin by different enzymes stain different colours with iodine (Kakefuda and Duke, 1984), so tentative identiﬁcation of starch-degrading activities is possible using this approach (Zeeman *et al.*, 1998); endo-amylases (such as α-amylases) create clear colourless bands, BAMs create brown bands, and debranching enzymes create pale blue bands. Whilst identiﬁcation of specific enzyme activities on this basis alone is not conclusive, we used this approach to highlight any differences between samples in terms of the complement of starch-degrading enzymes present rather than assigning bands to any specific starch-degrading enzymes.

### Data analysis

Where appropriate, data were analysed using the statistical software package SPSS. Before carrying out statistical tests, normality distribution (i.e. homogeneity) of the data was checked using the Kolmogorov–Smirnoff statistic (*P*>0.05). Means were compared using ANOVA.

## Results

### **Identification of the gene encoding the major chloroplastic** α **-glucan phosphorylase in *Kalanchoë fedtschenkoi***

Three *PHS* genes were identified in the two available genomes of *K. fedtschenkoi.* Multiple sequence alignment with the Arabidopsis *PHS* genes (Supplementary Fig. S1) and subsequent phylogenetic analysis (Supplementary Fig. S2) revealed that the gene named hereafter as *KfPHS1* (KfGene000193/Kaladp0024s0136.1) was the closest orthologue of the Arabidopsis chloroplast-targeted *PHS1* gene. The *KfPHS2* orthologue was also identified as sister of Arabidopsis cytosolic *PHS2* (Supplementary Fig. S2). ChloroP predicted a 56 amino acid residue N-terminal chloroplast transit peptide for the *KfPHS1* gene, but not for the *KfPHS2* gene (Supplementary Fig. S1). Furthermore, the predicted transit peptide within the *KfPHS1* gene aligned with the predicted N-terminal chloroplast transit peptide within the previously characterized, chloroplast-localized *PHS1* gene from Arabidopsis (Supplementary Fig. S1).

The genomes of both *K. fedtschenkoi* accessions contained a third *PHS* gene, named here as *KfPHS3*, which diverged strongly from *KfPHS1* and *KfPHS2* (Supplementary Fig. S2). ChloroP predicted a 47 amino acid N-terminal chloroplast transit peptide for *KfPHS3*, although this N-terminal region did not align with the chloroplast transit peptide region of Arabidopsis *PHS1* (Supplementary Fig. S1). Based on these findings, *KfPHS1* was selected here for targeted silencing using RNAi as it possessed a predicted chloroplast transit peptide, was the closest orthologue of the Arabidopsis chloroplast-localized *PHS1* gene, and was by far the most abundant *PHS* transcript detected in leaves performing CAM based on RNA-seq data for *K. fedtschenkoi* (Yang *et al.*, 2017).

The 357 bp *KfPHS1* nucleotide sequence region cloned into the hairpin RNA RNAi binary construct used to generate RNAi lines was 100% identical to the target transcript *KfPHS1* but shared much more limited sequence identity with *KfPHS2* and *KfPHS3* (Supplementary Fig. S3). Importantly, the longest contiguous runs of pairwise identity between the *KfPHS1* RNAi fragment and the corresponding regions of *KfPHS2* and *KfPHS3* were 12 bp and 9 bp, respectively (Supplementary Fig. S3). Thus, the *KfPHS1* RNAi construct was predicted to silence only the targeted *KfPHS1* transcripts, with no off-target effects.

### Isolation of RNAi mutant lines of *Kalanchoë fedtschenkoi*

**Fig. 2.**
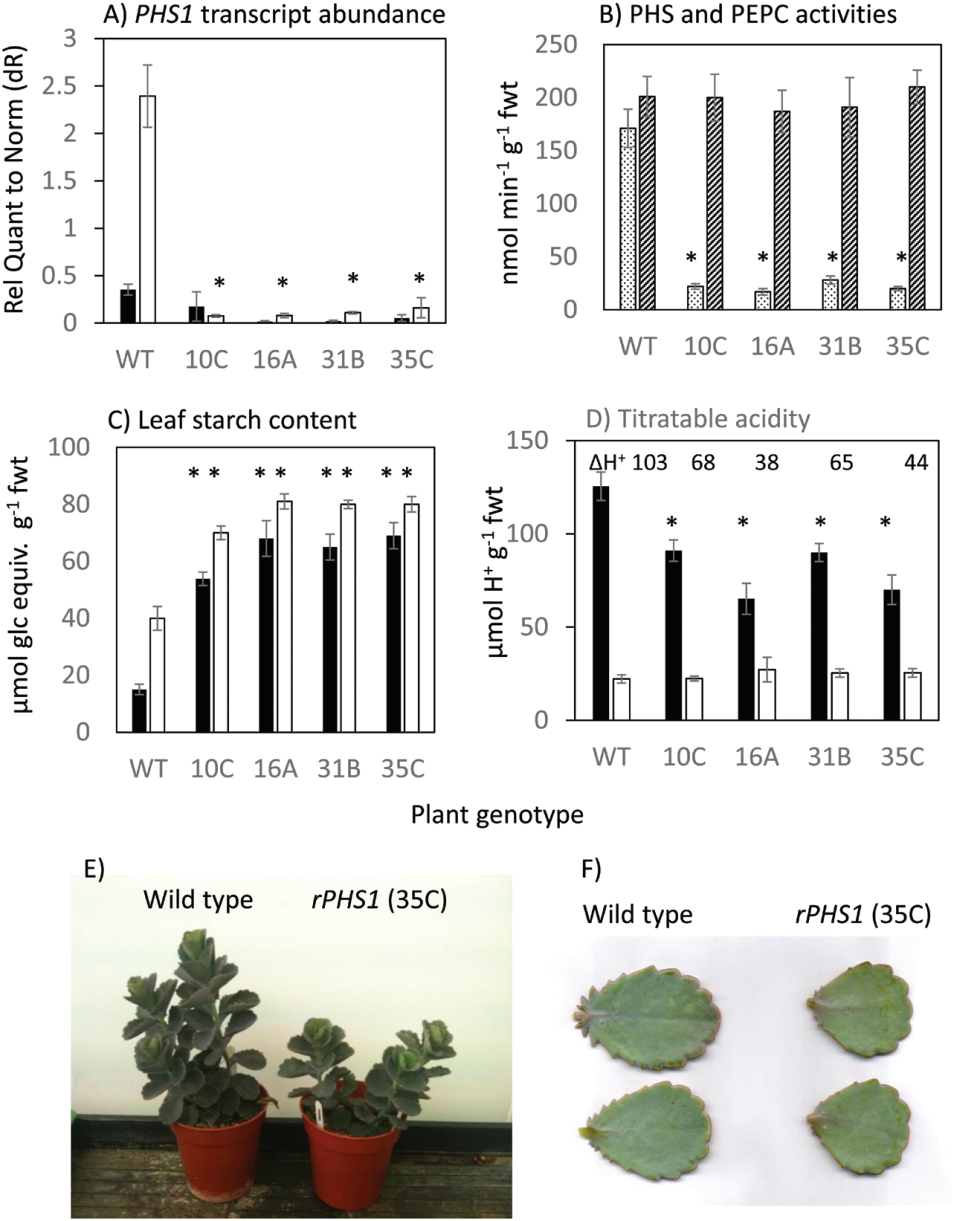
(A) Transcript abundance of *KfPHS1* (plastidic α-glucan phosphorylase) in the wild type and in four independent RNAi lines of the obligate CAM species *Kalanchoë fedtschenkoi* sampled at the end of the night (filled columns) and the end of the day (open columns). (B) Extractable activities of α-glucan phosphorylase (dotted fill) and phosphoenolpyruvate carboxylase (diagonal line fill) in the wild type and *rPHS1* lines, (C) Leaf starch content in the wild type and *rPHS1* lines sampled at the end of the night (filled columns) and the end of the day (open columns). (D) Leaf titratable acidities in the wild type and *rPHS1* lines sampled at the end of the night (filled columns) and the end of the day (open columns). Each point is the mean of four biological replicates ±SEM and where * indicates a significant difference (*P*<0.05) between the wild type and *rPHS1* lines. (E) Four-month-old plants and (F) leaf pair 6 (LP6) from plants raised under growth room conditions of 12 h photoperiod, day/night temperature of 25/19 °C, and PPFD at plant height of 250 µmol photons m^–2^ s^–1^.

Primary transformants were grown to the six leaf pairs stage and screened for changes in dawn and dusk leaf acidity, equating to CAM-mediated malic acid accumulation, and starch accumulation using a high-throughput screen of leaf discs sampled from LP6. From this screen, four independent *rPHS1* lines were selected and grown up to the 10 leaf pairs stage. RT–qPCR was used to measure *KfPHS1* transcript abundance sampled at the start and end of the photoperiod with primers outside the region targeted with the hairpin RNA RNAi construct. All four *rPHS1* lines showed a significant (*P*<0.05) reduction in abundance of the endogenous *KfPHS1* transcripts, which were >10-fold lower than in the wild type when sampled at the end of the photoperiod (Fig. 2A). Total extractable PHS activity at the end of the photoperiod was also significantly lower (~10-fold) in all four *rPHS1* lines when compared with the wild type, whilst PEPC activity was comparable across all genotypes (Fig. 2B).

Growth was significantly reduced in the *rPHS1* plants, illustrated for one representative line (Fig. 2E, F) and quantified for all four lines by reductions in shoot and root dry weights which were ~60% and 30%, respectively, of those in the wild type (*P*<0.05; Table 1). Leaf area was also reduced significantly (*P*<0.05), and leaves (LP6) from all *rPHS1* lines were ~20% more succulent in comparison with wild-type plants (*P*<0.05).

**Table 1.** Measured plant growth parameters for the wild type and four independent lines of *rPHS1*

	WT	Line 10C	Line 16A	Line 31B	Line 35C
Shoot dry weight (g)	4.88±0.51	3.11±0.26*	2.81±0.18*	3.18±0.31*	3.02±0.11*
Root dry weight (g)	1.03±0.07	0.46±0.04*	0.31±0.04*	0.42±0.03*	0.33±0.02*
Total leaf area (cm^2^)	43.8±4.1	37.0±4.0*	33.0±3.2*	35.1±3.9*	36.5±2.6*
Succulence (LP6) (kg m^–2^)	1.59±0.12	1.89±0.15*	1.97±0.12*	1.85±0.11*	1.92±0.14*

All data are shown as the mean of five independent biological replicates ±SEM and where * indicates a significant difference (*P*<0.05) between the wild type and *rPHS1* lines.

### Starch content and diel changes in titratable acidity

Leaf starch content was significantly (*P*<0.05) elevated relative to the wild type in all four *rPHS1* lines, consistent with an important functional role for *KfPHS1* in nocturnal starch degradation in *K. fedtschenkoi* (Fig. 2C). Overnight degradation of leaf starch was ~25 µmol glucose equivalents g^–1^ FW in the wild type, but this was reduced by ~50% across all the *rPHS1* lines. Diel changes in the nocturnal accumulation of titratable acidity due to CAM-associated dark CO_2_ fixation showed that the four *rPHS1* lines only accumulated ~50% of the acidity detected at dawn for the wild type (Fig. 2D). Titratable acidity is used as a proxy for nocturnal malic acid accumulation and storage in the mesophyll vacuole in CAM species because malic acid is by far the predominant organic acid present in CAM leaves, and 2 mol H^+^ are equivalent to 1 mol malic acid,

### Soluble sugar profiles

**Fig. 3.**
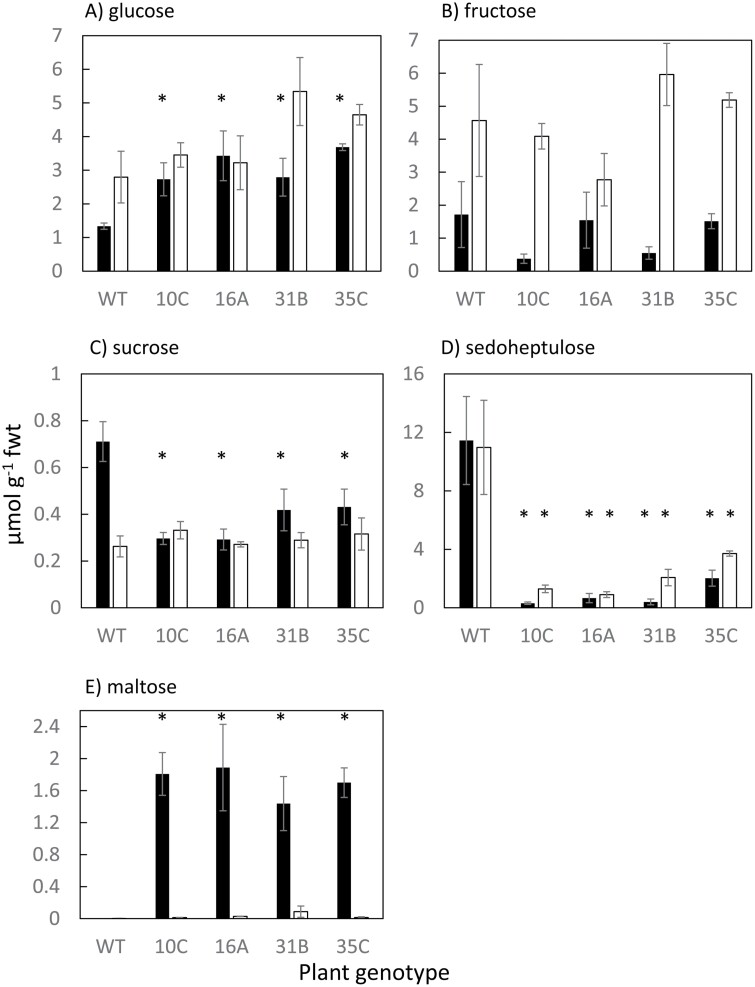
Soluble sugar profiles (A–E; µmol g^–1^ FW) in leaves of the wild type and four independent *rPHS1* lines sampled at the end of the night (filled columns) and the end of the day (open columns). Each point is the mean of four biological replicates ±SEM and where * indicates a significant difference (*P*<0.05) between the wild type and *rPHS1* lines.

The composition, abundance, and diel turnover of leaf soluble sugars were substantially altered in the *rPHS1* lines compared with the wild type (Fig. 3). Glucose content was significantly (*P*<0.05) elevated in all *rPHS1* lines sampled at the end of the night period when compared with the wild type (Fig. 3A), whereas sucrose and sedoheptulose contents were significantly lower (*P*<0.05) in *rPHS1* lines at the end of the night compared with the wild type (Fig. 3C, D). All of the independent *rPHS1* lines accumulated maltose to between 1.5 µmol g^–1^ FW and 2 µmol g^–1^ FW by the end of the dark period, whereas the wild type possessed a negligible maltose content at the end of both the light and dark periods (Fig. 3E).

### Leaf gas exchange and water use efficiency

**Fig. 4.**
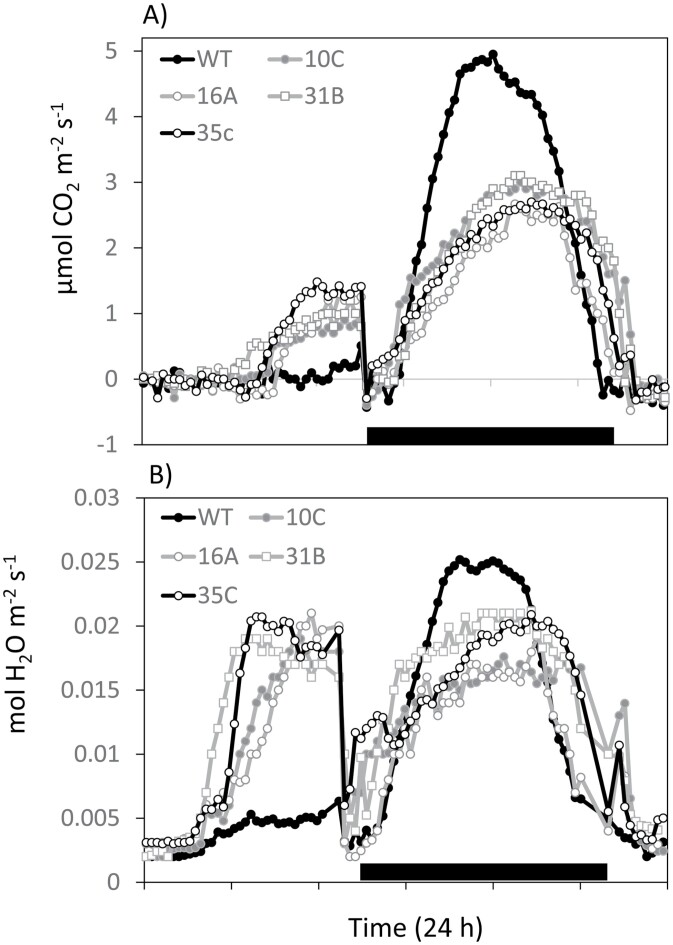
Diel changes in (A) net CO_2_ uptake and (B) stomatal conductance in the wild type and four independent *rPHS1* lines. The solid filled bar on the *x*-axis represents the period of darkness.

Diel profiles of net CO_2_ exchange revealed a significant reduction in nocturnal net CO_2_ uptake in all *rPHS1* lines that was ~55–65% of total nocturnal net CO_2_ uptake in the wild type (Fig. 4A; Table 2). The reduction in nocturnal net CO_2_ uptake in the *rPHS1* lines was consistent with the reduced nocturnal malate accumulation in these plants relative to the wild type, as revealed by the titratable acidity measurements (Fig. 2D). Whilst nocturnal CAM activity was suppressed in the *rPHS1* lines, light period net CO_2_ uptake was enhanced compared with the wild type, with considerable net CO_2_ uptake measurable over the last 4–5 h of the photoperiod in all *rPHS1* lines (Phase IV, Fig. 4A). When integrated over 24 h, total net CO_2_ uptake calculated on a leaf area basis was comparable in the wild type and the *rPHS1* lines (Table 2). In general, diel patterns of stomatal conductance tracked the trends described for the CO_2_ exchange, with enhanced daytime stomatal conductance in *rPHS1* lines compared with the wild type during Phase IV (Fig. 4B). Whilst the curtailed ability of *rPHS1* lines to degrade starch resulted in lower net CO_2_ uptake during the night compared with the wild type, maximal nocturnal values for stomatal conductance were less affected in the *rPHS1* lines compared with the wild type (Fig. 4A, B). Such data indicate that night-time stomatal opening is not solely determined by the magnitude of nocturnal carboxylation and draw-down in internal [CO_2_]. Water loss over 24 h was calculated by integrating the area under diel measurements of leaf transpiration and, together with comparable calculation of total net CO_2_ uptake over 24 h, we calculated the instantaneous WUE (Table 2). The *rPHS1* lines showed significantly reduced WUE both at night and over 24 h compared with the wild type.

**Table 2.** Integrated net CO_2_ uptake over the night and over the entire 24 h cycle for leaf pair 6 of the wild type and four independent lines of *KfPHS1* plants

Genotype	Integrated net CO_2_ uptake (mmol m^–2^)		Instantaneous water use efficiency (mmol CO_2_:mol H_2_O)	
	Night	24 h	Night	24 h
Wild type	109.3±8.6	104.6±19.4	12.7±0.4	8.7±0.8
Line 10C	70.8±9.1*	92.0±9.1	9.1±0.9*	5.4±0.6*
Line 16A	60.3±9.1*	81.1±14.1	9.3±0.8*	5.5±0.6*
Line 31B	68.1±9.4*	94.1±13.5	8.9±0.9*	5.1±0.4*
Line 35C	64.9±11.7*	89.4±10.9	8.2±1.2*	4.4±0.8*

Instantaneous water use efficiencies were calculated by integrating transpirational water loss over the night and over the entire 24 h cycle and then calculating the ratio of integrated net CO_2_ uptake to integrated net water loss. Data are shown as the mean of three independent biological replicates ±SEM and where * indicates a significant difference (*P*<0.05) between wild-type and *rPHS1* plants.

### Activity and transcript abundance of enzymes and transporters implicated in starch degradation

**Fig. 5.**
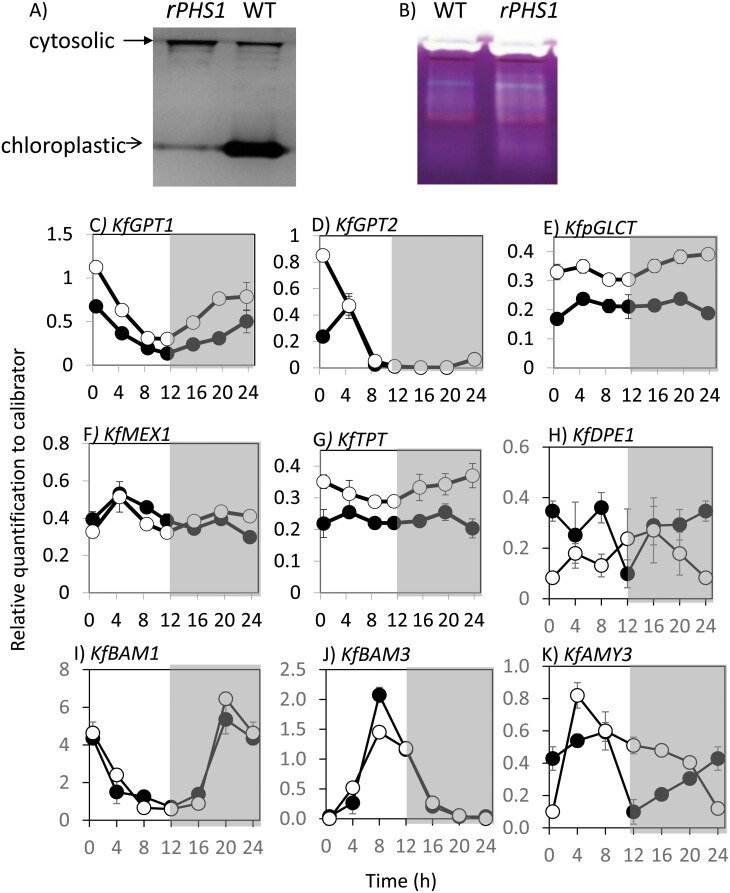
(A) Confirmation of reduced activity measured via native PAGE of plastidic α-glucan phosphorylase (PHS1) in the wild type (WT) and an RNAi line (line 35C, *rPHS1*). (B) In-gel activities of other enzymes implicated in starch degradation in both the WT and *rPHS1*, with prominent white bands typical of endoamylases (such as α-amylases), brown bands typical of β-amylases, and blue bands typical of debranching enzymes. (C–K) Diel changes in transcript abundance of other enzymes and transporters implicated in starch degradation in leaves of the WT (filled symbols) and *rPHS1* (open symbols). These included: (C) plastidic glucose-P translocator 1 (*KfGPT1*); (D) plastidic glucose-P translocator 2 (*KfGPT2*); (E) plastidic glucose transporter (*KfpGLCT*); (F) chloroplast maltose transporter (*KfMEX1*); (G) triose-P transporter (*KfTPT*); (H) chloroplastic disproportionating enzyme 1 (*KfDPE1*); (I) β-amylase 1 (*KfBAM1*); (J) β-amylase 3 (*KfBAM3*); and (K) α-amylase 3 (*KfAMY3*). Data presented are the mean of three technical replicates of each of three biological replicates and were normalized to the reference gene *KfTEDI*; error bars represent the SEM calculated for each biological replicate. The shaded area in each graph represents the period of darkness.

As all four of the independent *rPHS1* lines displayed remarkably consistent changes in growth and across multiple CAM and starch breakdown-associated traits (Figs 2–4; Tables 1, 2), a single representative *rPHS1* line, line 35C, was used for further phenotypic characterization. A detailed RNA time course experiment was carried out to examine the temporal regulation of transcripts associated with starch degradation. RT–qPCR confirmed the suppression of *KfPHS1* steady-state transcript levels in line 35C over the complete 12 h light/12 h dark cycle, whilst the transcript abundance of two core CAM pathway genes, namely *KfPPC1* and its dedicated, circadian clock-controlled protein kinase, *PEPC KINASE1* (*KfPPCK1*), were comparable in the wild type and *rPHS1* (Supplementary Fig. S4). Native PAGE confirmed a strong reduction in the activity of chloroplastic α-glucan phosphorylase compared with that of the wild type, with no detectable change in cytosolic starch phosphorylase (Fig. 5A). A range of coloured bands in the amylopectin-containing gels indicated the presence of different hydrolytic enzyme activities which were similar in the wild type and line 35C (Fig. 5B). Apart from PHS, measured *in vitro* activities of enzymes potentially implicated in starch degradation were comparable between line 35C and the wild type (Supplementary Table S2).

Diel changes in the transcript abundance of selected genes associated with the amylolytic route of starch degradation were also measured in order to establish whether or not perturbing the phosphorolytic route of starch breakdown influenced temporal transcriptional regulation of other enzymes/transporters implicated in starch breakdown. Diel patterns of abundance for BAM1 and BAM3 (*KfBAM1* and *KfBAM3*) and chloroplastic disporportionating enzyme (*KfDPE1*) were comparable with those of the wild type, but transcript abundance of α-amylase 3 (*KfAMY3*) was higher in *rPHS1* at dusk and throughout the first half of the dark period (Fig. 5H–K). Of the dedicated transporters potentially implicated in export of starch degradation products from the chloroplast to the cytosol, elevated transcript abundance across the entire diel cycle was noted for *KfGPT1* (responsible for movement of G6P across the chloroplast membrane), for *KfGlcT* (movement of glucose), and for *KfTPT* (movement of triose phosphates) in the r*PHS1* line compared with the wild type (Fig. 5C, E, G). Transcript abundances of the maltose transporter *KfMEX1* and a second *GPT* transcript (*KfGPT2*) were comparable at most of the sampled time points across the light/dark cycle in the wild type and *rPHS1* (Fig. 5D, F).

### Plasticity of CAM phases

**Fig. 6.**
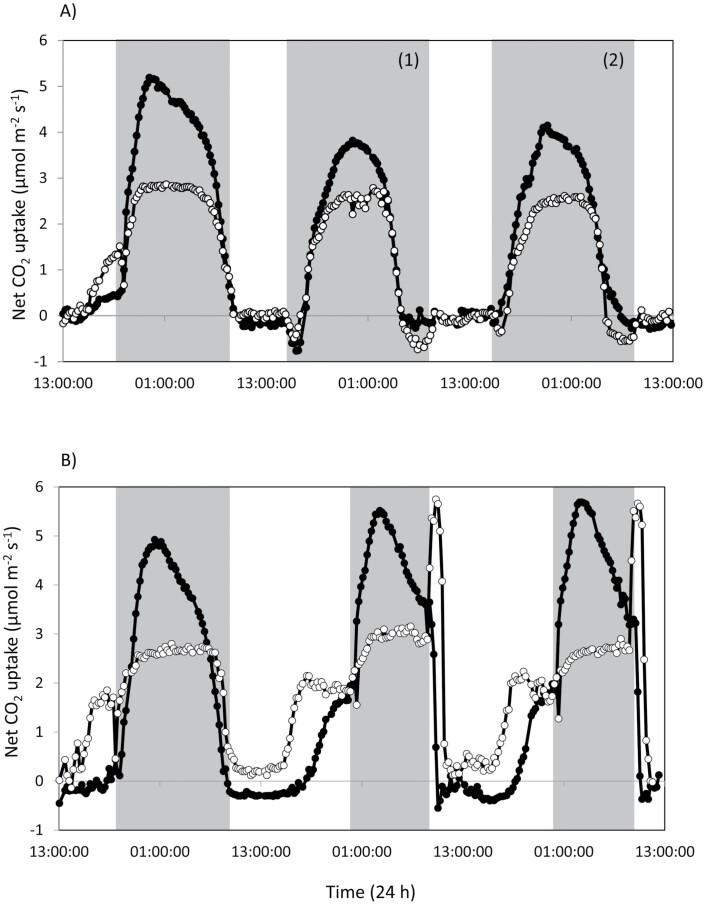
Diel changes in leaf net CO_2_ uptake in wild-type (filled circles) and *rPHS1* plants (open circles) under 12 h photoperiods and following transfer to: (A) 8 h photoperiods and (B) 16 h photoperiods. The shaded areas in graphs represent the periods of darkness.

To test the hypothesis that curtailing capacity for nocturnal starch degradation would compromise plasticity of the diel CAM phases, we compared patterns of net CO_2_ uptake in plants shifted from a 12 h photoperiod to a shortened 8 h photoperiod (Fig. 6A), or to an extended 16 h photoperiod (Fig. 6B). Integrated diurnal and nocturnal net CO_2_ uptake were also calculated (Table 3). *rPHS1* line 35C was compromised in its ability to adjust nocturnal CO_2_ uptake in response to an extended night/shortened 8 h photoperiod (Fig. 6A). After the second 8 h photoperiod, wild-type plants had started to extend the duration of nocturnal net CO_2_ uptake compared with the *rPHS1* line, and had recovered 80% of their total diel CO_2_ uptake (compared with that under a 12 h photoperiod) (Fig. 6A; Table 3). In contrast, after 2 d under an 8 h photoperiod, *rPHS1* line 35C had recovered <60% of its total 24 h net CO_2_ uptake measured under the 12 h photoperiod (Fig. 6A; Table 3). The *rPHS1* line also showed a net release of CO_2_ towards the end of each extended night, indicating that recapture of respiratory CO_2_ by PEPC was limited, perhaps by PEP deficiency.

**Table 3.** Integrated net CO_2_ uptake for whole plants of the wild type and *rPHS1* (line 35C) grown under 12 h photoperiods and subsequently shifted to 8 h photoperiods or 16 h photoperiods for 24 h (1) and 48 h (2); see also Fig. 6

	Integrated net CO_2_ uptake					
	Day		Night		24 h	
	WT	*KfPHS1*	WT	*KfPHS1*	WT	*KfPHS1*
12 h photoperiod	5±0.4	26±1.9*	156±17	106±9*	161±18	132±11
8 h photoperiod (1)	0	0	111±10	81±8*	111±10	81±8*
8 h photoperiod (2)	0	0	132±11	82±7*	132±11	82±7*
12 h photoperiod	2±0.3	26±3*	158±13	110±9*	160±12	136±12
16 h photoperiod (1)	35±3	84±7*	143±11	93±8*	178±15	177±15
16 h photoperiod (2)	25±2	76±5*	153±13	83±7*	178±15	159±13

Data are shown as the mean of three independent biological replicates ±SEM and where * indicates a significant difference (*P*<0.05) between the wild type and *rPHS1* plants.

Upon transfer to a longer photoperiod, wild-type plants increased maximum rates of nocturnal net CO_2_ uptake in the shorter dark period and augmented diel net CO_2_ exchange by enhancing daytime net CO_2_ uptake in Phase IV (Fig. 6B). After 48 h in the longer photoperiod, net diel CO_2_ exchange had increased by 11% over that in the 12 h photoperiod (Fig. 6B; Table 3). In contrast, the *rPHS1* line was unable to boost nocturnal net CO_2_ uptake in the shorter night and instead relied on enhanced daytime net CO_2_ uptake in Phases II and IV, which increased net diel CO_2_ exchange by 14% compared with that under the 12 h photoperiod (Fig. 6B; Table 3).

### Circadian rhythms in net CO_2_ uptake

**Fig. 7.**
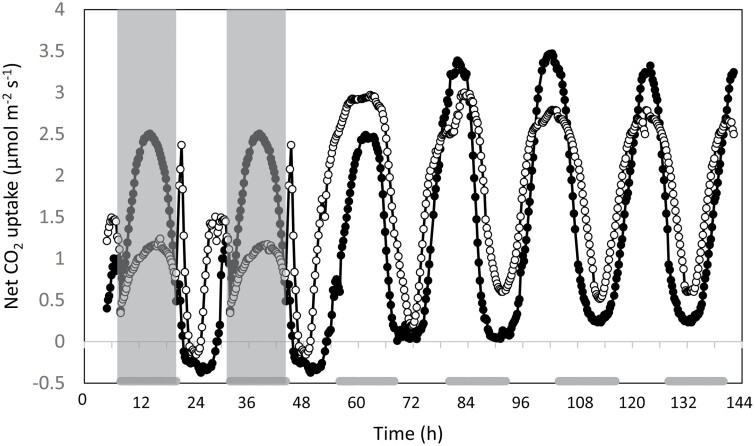
Diel changes in net CO_2_ uptake in wild-type (filled circles) and *rPHS1* plants (open circles) under two complete 24 h light/dark cycles and subsequently under constant PPFD (250 µmol m^–2^ s^–1^) and temperature (19 °C) for >95 h. The shaded areas in the graph represent the periods of darkness while plants were monitored in day/night cycles, and the grey blocks on the *y*-axis indicate when the dark period would have occurred when plants were monitored under constant environmental conditions.

To determine if curtailing starch degradation via the phosphorolytic route impacted the CAM-associated, persistent circadian rhythm of CO_2_ exchange observed under constant light and temperature (LL) conditions, we compared free-running rhythms of net CO_2_ exchange for whole plants of both the wild type and *rPHS1* line 35C (Fig. 7). Plants were initially measured for 48 h under the 12 h light/12 h dark conditions and then the growth chamber was switched to LL conditions and plants were monitored for 95 h under these free-running circadian conditions (Fig. 7). The data re-confirmed that this representative *rPHS1* 35C line displayed a significant reduction in whole-plant net carbon gain compared with the wild type over the initial 48 h of 12 h light/12 h dark cycles, with net CO_2_ fixation in the light period making a greater contribution to the 24 h carbon gain achieved by line 35C (Fig. 7). These findings replicated the data shown in Fig. 4A for all four independent *rPHS1* lines, which in turn emphasized that the subsequent LL data for line 35C (Fig. 7) were likely to be representative of the phenotype for the free-running circadian rhythm of CO_2_ exchange in all of the independent *rPHS1* lines. Following the release into LL free-running conditions, robust and high amplitude circadian rhythms of CO_2_ exchange were observed in both the wild type and line 35C, and rhythms remained robust for at least 100 h (Fig. 7). Over time, the peak of maximum CO_2_ fixation in the wild type shifted from the middle of the subjective dark period to the middle of the subjective light period. The *rPHS1* line also displayed a similar phase advance in the timing of the peaks in the rate of net CO_2_ exchange under free-running conditions. The period of net CO_2_ exchange circadian rhythm under LL at 19 °C was ~21 h and was similar for both genotypes. The amplitude of the circadian rhythm was greater for *rPHS1* in the first 24 h under LL, but the amplitude was greater for the wild type through the remainder of the LL run.

## Discussion

### Isolation of RNAi lines deficient in the major, chloroplast-localized α-glucan phosphorylase activity in an obligate CAM plant

It has previously been postulated that the requirement for nocturnal generation of PEP and the increased energetic demands of CAM relative to C_3_ photosynthesis might have resulted in a re-routing of chloroplastic starch degradation from the hydrolytic route to the phosphorolytic route (Borland *et al.*, 2016; Shameer *et al.*, 2018). To test this hypothesis, four independent RNAi lines of the obligate CAM species *K. fedtschenkoi* with a >10-fold reduction in the steady-state transcript abundance of chloroplastic α-glucan phosphorylase (*KfPHS1*) were isolated and subjected to detailed phenotypic characterization with respect to CAM and starch degradation-associated traits. All four independent *rPHS1* lines showed diminished nocturnal starch degradation and higher basal levels of starch throughout the 24 h diel cycle compared with the wild type (Fig. 2C), indicating that enzyme activity encoded by *KfPHS1* plays a central role in nocturnal starch degradation during CAM. Curtailment in nocturnal starch degradation was accompanied by reduced CAM activity in all lines, as indicated by diminished nocturnal acid accumulation and dark net CO_2_ uptake (Figs 2D, 4A). Extractable activities of the primary nocturnal carboxylase PEPC (Fig. 2B), and the diel regulation of transcript abundance of its activator, *KfPPCK1* (Supplemntary Fig. S4), were comparable in the wild type and *rPHS1*, so the reduced CAM activity observed in the *rPHS1* lines was consistent with the view that nocturnal starch degradation is a limiting factor for nocturnal carboxylation capacity during CAM (Borland and Dodd, 2002; Cushman *et al.*, 2008; Haider *et al.*, 2012).

A prominent role for the phosphorolytic route of starch degradation to provision substrates for nocturnal CO_2_ fixation via PEPC during CAM is in marked contrast to the situation in C_3_ plants such as *A. thaliana* and potato, where elimination or reduction of the plastid-localized isoforms of starch phosphorylase did not alter starch degradation or plant growth (Sonnewald *et al.*, 1995; Zeeman *et al.*, 2004). The phosphorolytic route of starch degradation in C_3_ plants has been proposed to provide carbon for metabolism inside the chloroplast by feeding substrate into the pentose phosphate pathway, particularly under conditions of stress when photorespiration is elevated (Zeeman *et al.*, 2004, 2007; Weise *et al.*, 2006). Expression of the chloroplast envelope-localized *GPT* has generally been shown to be restricted to heterotrophic non-green tissues in non-CAM species, suggesting limited export of G6P across the C_3_ chloroplast envelope (Kammerer *et al.*, 1998; Niewiadomski *et al.*, 2005). Four genes encoding *KfGPT* are present in the *K. fedtschenkoi* genome (Yang *et al.*, 2017). Transcripts of the two most abundant *KfGPT* transcripts in CAM-performing LP6 (*KfGPT1* and *KfGPT2*) were detected and found to oscillate in abundance over the 12 h light/12 h dark cycle in CAM LP6 of *K. fedtschenkoi* (Fig. 5C, D), consistent with previous reports for orthologous *GPT* genes in other CAM species (Kore-eda *et al.*, 2013; Borland *et al.*, 2016). The dawn-phased transcript peak for both *KfGPT* genes was ~12 h out of phase with that of transcripts encoded by *KfPHS1* in *K. fedtschenkoi*, which peaked 3.5 h before dusk (Supplementary Fig. S4A).Together, these results suggest either transcriptional (*KfPHS1*) or, perhaps, post-transcriptional regulation (*KfGPT*) of flux through the phosphorolytic route of starch breakdown. Alternatively, GPT could function in the early light period, potentially facilitating G6P import into the chloroplast as part of the recycling pathway that processes the pyruvate from malate decarboxylation in the light period back to leaf starch (Hartwell *et al.*, 2016). In comparison with the wild type, *KfGPT1* transcripts were induced across the diel cycle, and a 4-fold induced early peak was observed for *KfGPT2* specifically at dawn, in *rPHS1* line 35C (Fig. 5C, D). These data suggest the possibility of metabolic feedback control of *GPT* steady-state transcript abundance in the absence of *KfPHS1*, which could represent a futile compensatory mechanism aimed at enhancing transport of G6P across the chloroplast envelope when production of this metabolite is curtailed due to the loss of *PHS1*.

### Amylolytic degradation of starch in *rPHS1* plants

Despite the substantial knockdown of *KfPHS1* across all four independent lines, starch degradation and nocturnal net CO_2_ uptake were not abolished and were maintained at ~50% of the levels detected in the wild type (Figs 2C, 4A). This suggests a re-routing and/or enhancement of starch degradation through the amylolytic/hydrolytic pathway. Enhanced nocturnal production of maltose at night was detected in all four *rPHS1* lines, which is consistent with enhanced flux through the amylolytic pathway compared with the wild type, which had no detectable maltose (Fig. 3E). BAMs are the key amylolytic enzymes responsible for liberating maltose from the non-reducing ends of α1,4-linked glucan chains (Streb and Zeeman, 2012; Fig 1A). High extractable activities of BAM (Supplementary Table S2) and comparable abundance and diel cycling of transcripts encoding the catalytically active, chloroplast-localized *KfBAM1* and *KfBAM3* were found in both the wild type and line 35C (Fig. 5I, J), suggesting sufficient BAM capacity for nocturnal starch degradation in both lines of *K. fedtschenkoi*. In both the wild type and line 35C, activity *in vitro* of the cytosolic disproportionating isoform (DPE2) was low compared with *A. thaliana* (Chia *et al.*, 2004). In *A. thaliana*, DPE2 is critical for processing maltose, exported from the chloroplast during nocturnal starch degradation, into sucrose in the cytosol (Supplementary Table S2; Chia *et al.*, 2004). Low DPE2 activity (as measured *in vitro*) appears to be a characteristic of CAM species (Borland *et al.*, 2016; Ceusters *et al.*, 2019). Thus, in the absence of the phosphorolytic pathway of starch degradation in the *rPHS1* lines reported here, DPE2 is likely to present a bottleneck for the processing of maltose produced via amylolytic starch degradation. Indeed, the *rPHS1* plants were characterized by much lower glucose–maltose ratios at the end of the night compared with the wild type (Fig. 3A, E), indicating a restriction in sucrose synthesis, and reduced sedoheptulose content (Fig. 3D) also indicated a disruption to metabolic homeostasis in the *rPHS1* lines (Ceusters *et al.*, 2013). In common with other CAM species (Borland *et al.*, 2016), low transcript abundance was also noted for the chloroplast maltose transporter gene *KfMEX1* in both the wild type and the *rPHS1* line, which could present a further potential bottleneck for amylolytic processing of starch via maltose at night in *K. fedtschenkoi*.

Increased production of glucose from amylolytic starch breakdown and export from the chloroplast via the plastidic glucose transporter (*KfpGlcT*) could aid in alleviating the bottleneck in maltose processing in the *rPHS1* lines. All four *rPHS1* lines accumulated more glucose overnight compared with the wild type (Fig. 3A), and line 35C showed elevated transcript abundance of *KfpGlcT*, particularly in the dark period and at dawn (Fig. 5E). A potential route for enhancing amylolytic production of glucose in the *rPHS1* lines was suggested by altered diel patterns of *KfAMY3* transcripts, which showed elevated abundance relative to the wild type at the end of the light period and over the first half of the dark period in the RNAi line (Fig. 5K). In *A. thaliana*, AMY3 is a catalytically active chloroplastic α-amylase that can release a variety of branched and linear oligosaccharides from starch degradation (Streb and Zeeman, 2012). In the absence of *KfPHS1*, the linear oligosaccharides generated by AMY3 could be processed by chloroplastic DPE1, which showed greatly elevated activity (measured *in vitro*) in *K. fedstchenkoi* as well as in other CAM species when compared with Arabidopsis (Supplementary Table S2; Borland *et al.*, 2016; Ceusters *et al.*, 2019). Whilst AMY3 does not generally play a prominent role in nocturnal starch degradation in Arabidopsis, in cases where other enzymes implicated in starch degradation are missing, or under water deficit and osmotic stress, AMY3 can have a significant impact on starch degradation (Delatte *et al.*, 2006; Thalmann and Santelia, 2017). Recent characterization of the leaf proteome of *K. fedstchenkoi* indicated significantly higher protein abundance of AMY3 compared with BAM1 or BAM3 (Abraham *et al.*, 2020). In CAM plants, AMY3 could help to increase the overall rate of starch degradation (Weise *et al.*, 2011) and/or to provide longer glucan chain substrates that are more amenable to degradation by PHS1 (Shimomura *et al.*, 1982). The data presented here are an important indicator of how an intricate network of metabolic steps offers plasticity for modulating starch degradation to generate alternative substrates to fulfil different metabolic requirements. Further work is required to establish if perturbing specific enzymes and transporters implicated in the amylolytic route of starch degradation has an impact on the operation of CAM.

### Impacts of perturbing phosphorolytic starch degradation on carbon balance, water use, and photosynthetic plasticity

Despite a 50% reduction in night-time net CO_2_ uptake compared with the wild type in LP6, all *rPHS1* lines showed enhanced net CO_2_ uptake in the light period (Fig. 4A), with the result that 24 h carbon gain under 16 h light/8 h dark and 12 h light/12 h dark cycles was similar in LP6 for both the wild type and *rPHS1* (calculated on a leaf area basis; Table 2 and 3). Enhanced light period net CO_2_ uptake in the *rPHS1* lines was accompanied by higher stomatal conductance in the light (Fig. 4B), which increased transpirational water loss compared with the wild type. The *rPHS1* lines also showed lower WUE at night compared with the wild type since net CO_2_ uptake was suppressed to a greater extent than stomatal conductance (Table 2). Thus, 24 h WUE was 50% lower across all *rPHS1* lines compared with the wild type. Despite comparable diel carbon balances in LP6 noted between the wild type and all of the RNAi lines, the *rPHS1* plants showed a marked growth penalty, probably as a consequence of a lower leaf area index (Table 1). Thus, silencing the phosphorolytic route of starch degradation in *K. fedtschenkoi* impacted negatively on growth and WUE of this obligate CAM species.

The temporal separation of carboxylases that defines CAM engenders plasticity for modulating the duration and amplitude of the four phases of CAM in response to changing environmental conditions (Osmond, 1978; Ceusters *et al.*, 2008, 2010). In addition to this plasticity of the four phases being evident for all four independent *rPHS1* lines, representative line 35C displayed a compromised ability to modulate its net nocturnal CO_2_ uptake in response to a sudden change in the length of the photoperiod. In contrast to the wild type, *rPHS1* 35C was unable to boost nocturnal CO_2_ fixation via PEPC when moved from a 12 h dark period to an 8 h dark period (Fig. 6A; Table 3). Furthermore, in response to a switch from a 12 h dark to a 16 h dark period, line 35C was unable to prolong its nocturnal CO_2_ fixation, as observed for the wild type (Fig. 6B; Table 3). Since there was no evidence that the *rPHS1* line was compromised in PEPC activity/capacity (Fig. 2B), these data support the proposal that the *rPHS1* line lacked the ability to adjust the rate of starch breakdown to a change in the length of the night. In the C_3_ model species *A. thaliana*, it has been shown that the endogenous circadian clock matches starch breakdown to the anticipated length of the dark period (Stitt and Zeeman, 2012). In terms of the circadian rhythm of CAM-associated gas exchange in *K. fedtschenkoi*, both the wild type and *rPHS1* line 35C displayed the characteristic robust, high-amplitude, short-period, free-running oscillations of CO_2_ fixation for at least 100 h under continuous light and temperature (LL; Fig. 7), which is typical for *K. fedtschenkoi* (Wilkins, 1984, 1992; Boxall *et al.*, 2017, 2020). These data suggest that operation of the core circadian clock was not compromised in the *rPHS1* line, at least not with respect to the core clock mediating temporal control and optimization of the CAM-associated CO_2_ fixation rhythm. Moreover, the robust circadian rhythm of CO_2_ exchange under LL displayed by the *rPHS1* line revealed that preventing nocturnal starch degradation via the chloroplast-localized α -glucan phosphorylase encoded by *KfPHS1* did not impact as negatively on the circadian clock control of CO_2_ fixation as other genetic manipulations of the CAM pathway that prevented malate decarboxylation and/or pyruvate recycling in the light (Dever *et al.*, 2015) or PEPC phosphorylation/activity in the dark (Boxall *et al.*, 2017, 2020). Thus, the phosphorolytic route of starch degradation does not appear to be under the central circadian regulation that controls dark carboxylation and daytime decarboxylation in *K. fedstchenkoi*. In terms of CAM evolution, these findings present the hypothesis that the re-routing of starch degradation from the hydrolytic to the phosphorolytic route occurred as a downstream consequence of changes in the diel control of PEPC-mediated carboxylation.

Previous work on *A. thaliana* has shown that interrupting sugar export from leaves, or feeding sugars to leaves in the dark, leads to slower breakdown of starch (Stitt and Zeeman, 2012). An analogous scenario in terms of sugar accumulation impacting starch turnover could be linked to maltose hyperaccumulation in the *rPHS1* lines (Fig. 3E). Thus, perturbed sugar homeostasis in the RNAi lines impacted the rate of starch breakdown which in turn curtailed plasticity for modulating nocturnal starch degradation and dark CO_2_ uptake in response to changes in the supply and demand for carbon.

Although the *rPHS1* plants were compromised in adjusting dark CO_2_ uptake in response to changing metabolic demands, plasticity for enhancing light period net CO_2_ uptake was less hampered. This was particularly marked in the circadian experiments, where, following transfer to LL conditions, the *rPHS1* line showed a doubling of 24 h net CO_2_ uptake, particularly during the first cycle (Fig. 7). Rhythmicity of CAM gas exchange under LL appears to involve interplay of both C_3_ and C_4_ carboxylases (Davies and Griffiths, 2012). Since PEPC capacity was comparable in both the wild type and the RNAi line, it appears that the *rPHS1* line was able to maintain carbon balance under changing environmental conditions by invoking a greater contribution from direct Rubisco-mediated net CO_2_ uptake, but with the consequence of greater transpirational water loss.

In conclusion, our data show that the phosphorolytic pathway of starch degradation, which plays a minor role in Arabidopsis, is critical for efficient operation of the CAM cycle and leads to optimized growth and WUE by provisioning PEP for nocturnal CO_2_ fixation via PEPC. These findings indicate a fundamental difference in the route of starch degradation between species and have clear relevance for ongoing efforts to engineer CAM into non-CAM species as a means of boosting crop WUE for a warmer, drier future.

## Supplementary data

The following supplementary data are available at *JXB* online.

Fig. S1. Sequence alignments of *KfPHS* genes with Arabidopsis orthologues.

Fig. S2. A Neighbor–Joining consensus tree confirmed that *KfPHS1* was the closest *K. fedtschenkoi* orthologue of the known Arabidopsis chloroplast-localized *PHS* gene *AtPHS1.*

Fig.S3. Confirmation of the gene specificity of the 357 bp *KfPHS1* region used in the hairpin RNA RNAi binary construct designed to target the silencing of *KfPHS1*.

Fig. S4. Diel changes in transcript abundance of plastidic α-glucan phosphorylase, phosphoenolpyruvate carboxylase (*KfPEPC/KfPPC1*) and phosphoenolpyruvate carboxylase kinase1 (*KfPPCK1*) in leaves of wild-type and *rPHS1* line 35C plants.

Table S1. List of forward and reverse primers used in RT–qPCR.

Table S2. Extractable activities of starch-degrading enzymes measured *in vitro* for both wild-type and *rPHS1* plants sampled at the end of the photoperiod.

erab132_suppl_Supplementary_Material

## Data Availability

All data supporting the findings of this study are available within the paper and within the supplementary data published online.
