# Supplementary material for: Phosphorolytic degradation of leaf starch via plastidic α-glucan phosphorylase leads to optimized plant growth and water use efficiency over the diel phases of Crassulacean acid metabolism
Source: J Exp Bot. 2021 Mar 22;72(12):4419–34. doi: 10.1093/jxb/erab132 (PMC8266541; doi:10.1093/jxb/erab132)
Supplement: erab132_suppl_Supplementary_Material [file erab132_suppl_supplementary_material.pdf]

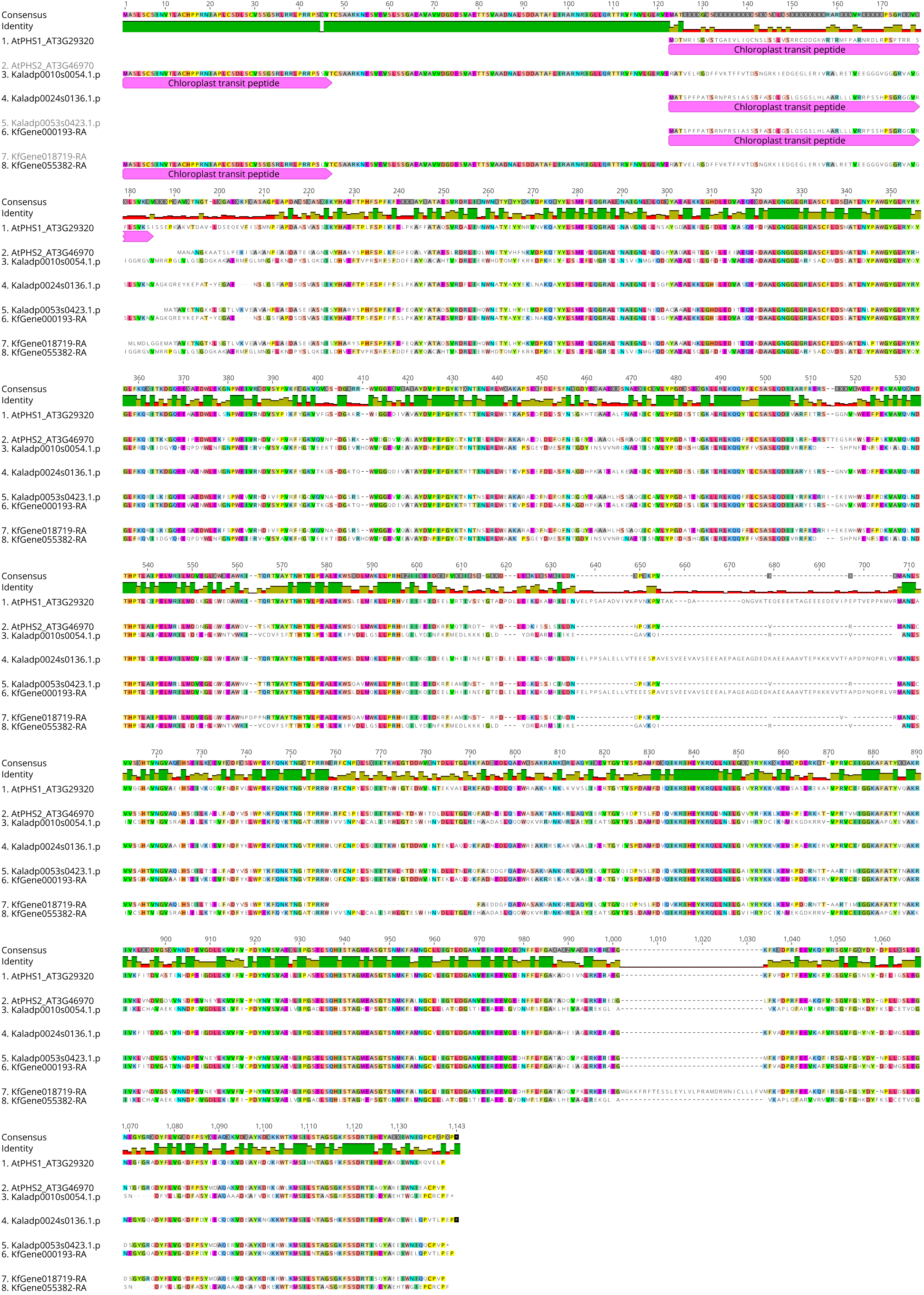
Consensus
Identity
1. AtPHS1_AT3G29320
2. AtPHS2_AT3G46970
3. Kaladp0010s0054.1.p
4. Kaladp0024s0136.1.p
5. Kaladp0053s0423.1.p
6. KfGene000193-RA
7. KfGene018719-RA
8. KfGene055382-RA
Chloroplast transit peptide

Supplementary Figure 1: The *KfPHS1* gene silenced with RNAi in this study possesses a predicted chloroplast transit peptide that aligns with the corresponding transit peptide of the Arabidopsis chloroplast–targeted *PHS1* gene AT3G29320. The full length predicted amino acid sequences for the 2 *PHS* genes found in the genome of *Arabidopsis thaliana* and the three *PHS* genes found in the *Kalanchoë fedtschenkoi* genomes were aligned using the software Geneious with the "Geneious Align" multiple protein sequence alignment algorithm. The chloroplast transit peptides predicted by ChloroP were annotated onto each sequence with the magenta arrows below the corresponding predicted transit peptide sequence. *K. fedtschenkoi* sequences from the two distinct accessions or ecotypes for which complete genome sequences are available are included, but KfGene000193 is the specific *KfPHS1* gene silenced in the Kew/Glasgow/Liverpool accession/ecotype used in this study. The *KfPHS1* gene shares a predicted N-terminal chloroplast transit peptide with the Arabidopsis chloroplast-localized *PHS1* (AT3G29320). The sequences aligned were: AT3G39320 *PHS1* (chloroplast-localized *PHS1*), AT3G46970 (cytosolic *PHS2*), KfGene000193/Kaladp0024s0136.1.p (chloroplast-localized *KfPHS1*), KfGene018719/Kaladp0053s0423.1.p (cytosolic *KfPHS2*) plus a further predicted chloroplast-localized *KfPHS3* gene, KfGene055382/Kaladp0010s0054.1.p. This novel, chloroplast-localized *KfPHS3* gene, which did not have an ortholog in Arabidopsis, was not targeted in this study due to it displaying low relative transcript abundance when compared to *KfPHS1* in mature, CAM-performing leaves of *K. fedtschenkoi* (Yang *et al*., 2017).

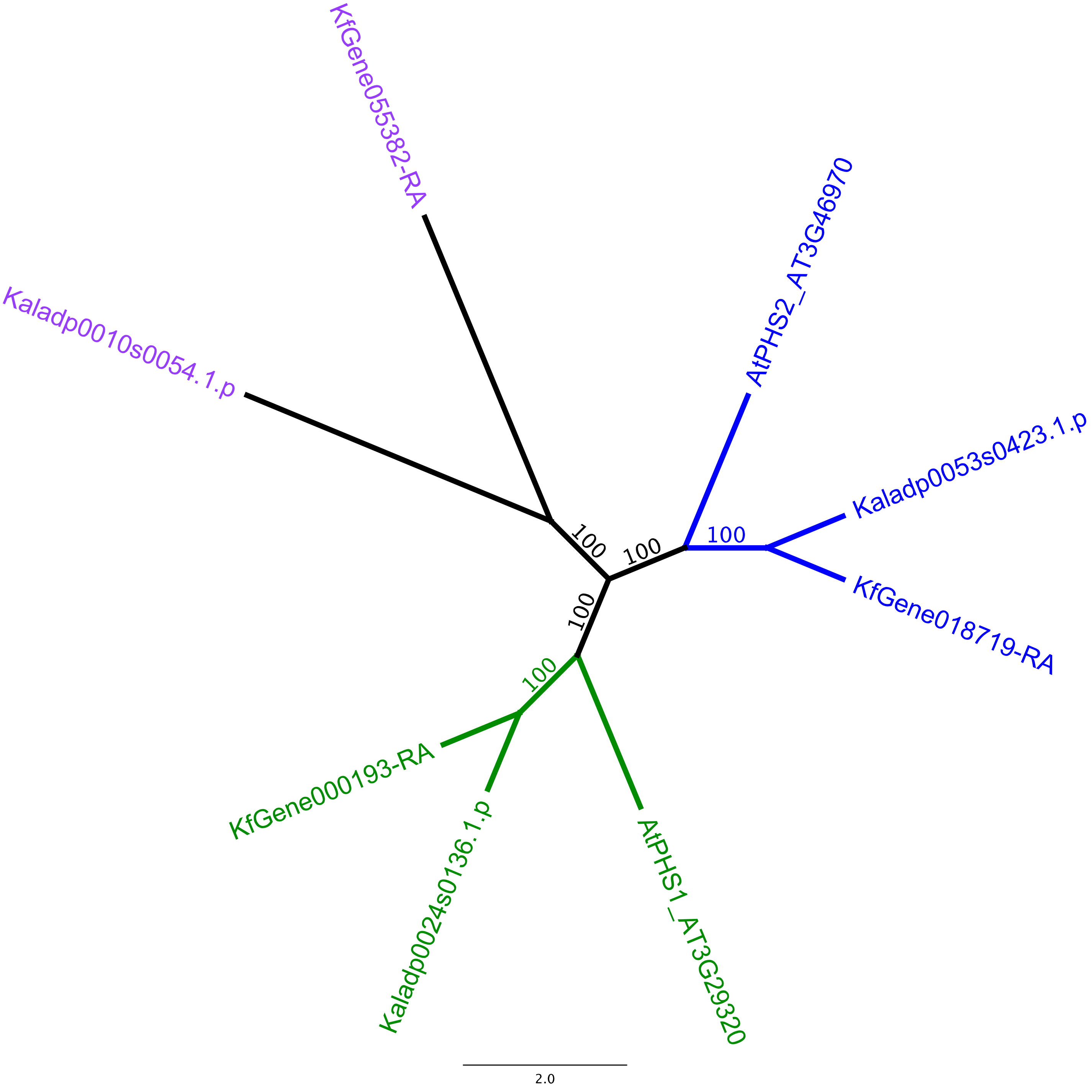

Supplementary Figure 2: A neighbour-joining consensus tree confirmed that *KfPHS1* (KfGene000193/Kaladp0024s0136.1) was the closest *K. fedtschenkoi* ortholog of the known Arabidopsis chloroplast-localized *PHS* gene *AtPHS1* (AT3G29320). Chloroplast localized *AtPHS1* and the orthologous sequence in the *K. fedtschenkoi* genomes, KfGene000193/Kaladp0024s0136.1, are highlighted in green, the cytosolic *AtPHS2* and orthologous *K. fedtschenkoi* sequences, KfGene018719/Kaladp0053s0423.1, are blue, and the third PHS gene in the *K. fedtschenkoi* genomes, *KfPHS3* (KfGene055382/Kaladp0010s0054.1), labelled in purple. Note that there is no ortholog of *KfPHS3* in the Arabidopsis genome. The multiple sequence alignment shown in supplementary figure 1 was used to generate a neighbour-joining tree with the tree building option of the bioinformatics software Geneious, and the Jukes-Cantor genetic distance matrix. 1000 bootstraps were used, and the consensus tree is displayed. The branch labels show the % consensus support. The scale bar shows the degree of sequence divergence (%) represented in the length of the individual branches of the tree.

a)

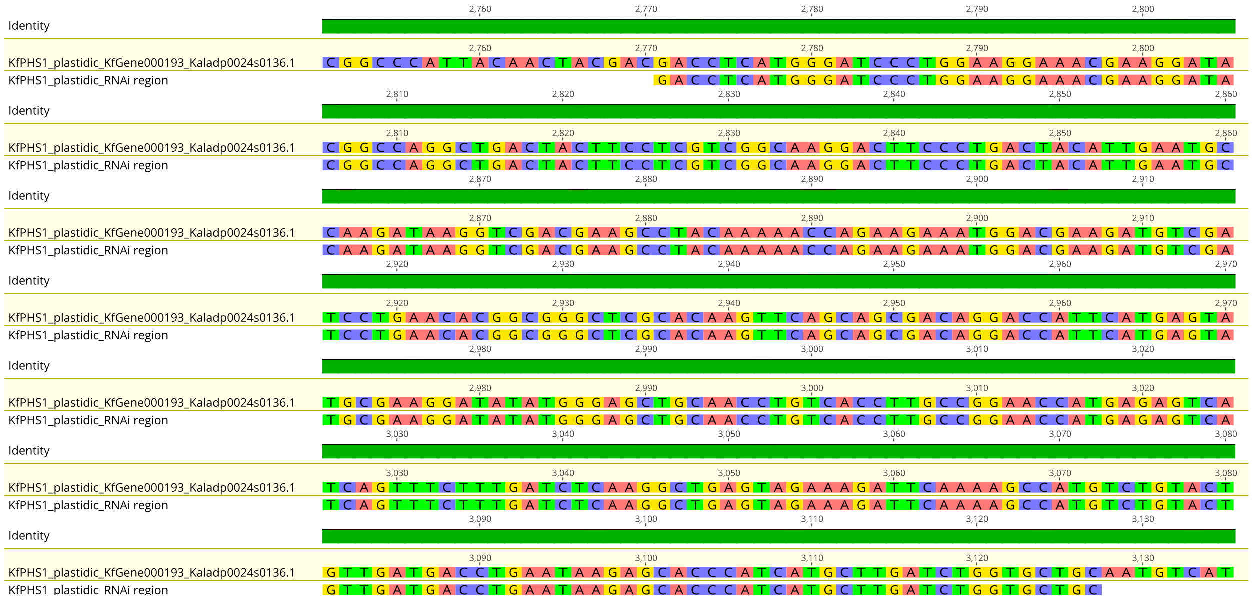
Identity
KfPHS1_plastidic_KfGene000193_Kaladp0024s0136.1
KfPHS1_plastidic_RNAi region

b)

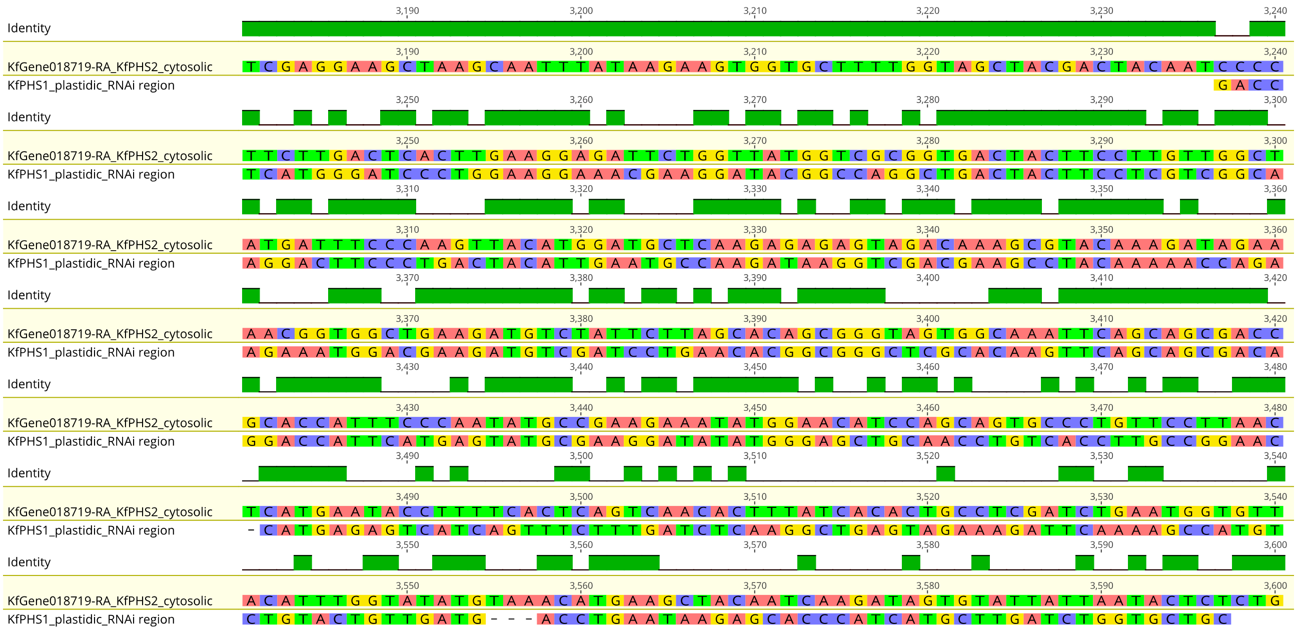
Identity
KfGene018719-RA_KfPHS2_cytosolic
KfPHS1_plastidic_RNAi region

c)

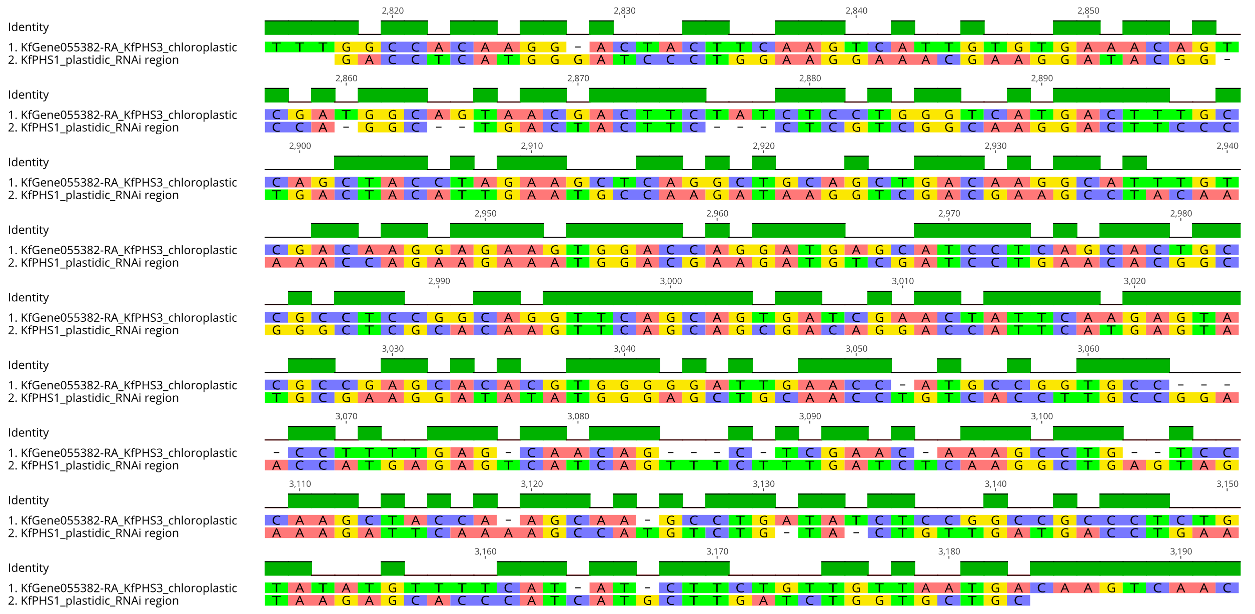
Identity
1. KfGene055382-RA_KfPHS3_chloroplastic
2. KfPHS1_plastidic_RNAi region

Supplementary Figure 3: Confirmation of the gene specificity of the 357 bp *KfPHS1* region used in the hairpin RNA RNAi binary construct designed to target the silencing of *KfPHS1*. **a)** pairwise alignment of the *KfPHS1* RNAi region to the corresponding region of the targeted *KfPHS1* transcript. **b)** pairwise alignment of *KfPHS2* to the *KfPHS1* RNAi fragment. **c)** pairwise alignment of *KfPHS3* to the *KfPHS1* RNAi fragment. Pairwise nucleotide alignments were generated using the bioinformatics software Geneious and its in-built “Geneious Align” option for the nucleotide sequences of each of the three *PHS* genes in the *K. fedtschenkoi* genomes, namely chloroplastic *KfPHS1* (KfGene000193/Kaladp0024s0136.1), cytosolic *KfPHS2* (KfGene018719/Kaladp0053s0423.1) and chloroplastic *KfPHS3* (KfGene055382/Kaladp0010s0054.1). The pairwise nucleotide identity is highlighted with the continuous green bar above the sequence alignments. Note that whilst, as expected, the entire 357 bp RNAi region displays 100% pairwise identity to *KfPHS1* in a), the stretches of 100% pairwise nucleotide identity between *KfPHS2* or *KfPHS3* and the *KfPHS1* RNAi fragment in **b)** and **c)** are at most 12 bp and 9 bp long, respectively. During activation of the endogenous RNAi gene silencing pathway by the double stranded hairpin RNA (dsRNA) fragment generated from the *KfPHS1* RNAi transgene, the dsRNA will be digested by DICER into 21 bp fragments that are loaded onto the RISC complex. This in turn leads to gene specific degradation of the endogenous *KfPHS1* transcripts, but the alignments in b) and c) to *KfPHS2* and *KfPHS3* demonstrate that the *KfPHS1* RNAi fragment would not direct off-target degradation of any *KfPHS2* and *KfPHS3* transcripts.

Supplementary Figure 4

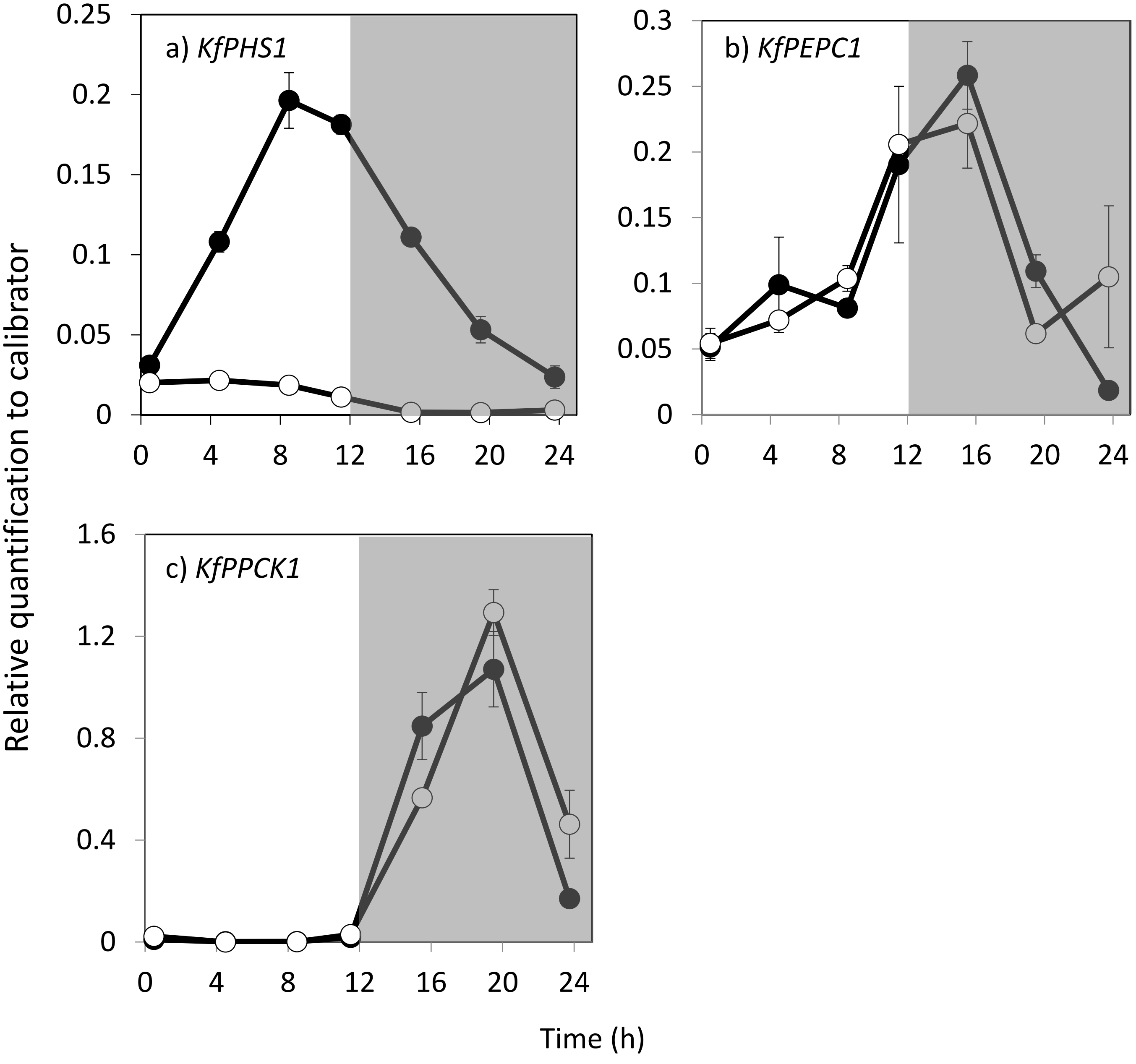

Supplementary Figure 4: Diel changes in transcript abundance of (a) plastidic α-glucan phosphorylase (KfPHS1), (b) phosphoenolpyruvate carboxylase (KfPEPC/KfPPC1) and (c) phosphoenolpyruvate carboxylase kinase1 (KfPPCK1) in leaves of wild type (solid circles) and rPHS1 line 35C plants (open circles). The shaded area in each graph represents the period of darkness. Data presented are the mean of 3 technical replicates of each of 3 biological replicates and were normalized to reference gene KfTEDI; error bars represent the standard error of the mean calculated for each biological replicate. The shaded area in each graph represents the period of darkness.

Supplementary Table 1.

List of forward and reverse primers used in qRT-PCR

PEPC F 5’ CAC AGC AGC TCC TTC CTC GC 3’
PEPC R 5’ ATG CGG AGG AAA TTG CTG GGG 3’

PPCK1 F 5’ TAC AGA GAG ACA GAG AGC AGG 3’
PPCK1 R 5’ CAT CTC CCA TCA CCA TCC ATC 3’

PHS1 F 5’ GCC ATG TCT GTA CTG TTG ATG AC 3’
PHS1 R 5’ GCA GCA CCA GAT CAA GCA TG 3’

MEX1 F 5’ GTG AAA GCT GCA AGG ACA GC 3’
MEX1 R 5’ GGA GTC GCC AAT AGC CAG TC 3’

TPT F 5’ GGA ATC CGA TCC TGG GAG TG 3’
TPT R 5’ AGA AGC AGT GCT GGA TGA GAG 3’

GLCT F 5’ ACC CAC GAA GTC AGC TTC TAC 3’
GLCT R 5’ GGT GAC AAA TGA TCA AGT CTT TTC C 3’

GPT1F 5’ GTA CCA GGT GCC AGT GGT AG 3’
GPT1 R 5’ CAA CAC GCT GCA CTT ACG TC 3’

GPT2 F 5’ GTC TGG TAG AGG TCA CAA GCC 3’
GPT2 R 5’ CGG GTA GGC TCA CAG TGA AC 3’

AMY3 F 5’ AGC CTC CAG CCA TCA TAA CC 3’
AMY3 R 5’ TGA TCA CTC ACA GGC CTT CG 3’

BAM1 F 5’ TGC TTT GCC TCG CTA TGA TG 3’
BAM1 R 5’ TTT CTC TCG CTT CCC CAC TG 3’

BAM3 F 5’ CCA GCA TGC AAA CTG TTC TCC 3’
BAM3 R 5’ TCC CCA GCA AGT TCT GTT CC 3’

BAM9 F 5’ AAA GGT CCA TCC CAG GCA AG 3’
BAM9 R 5’ TTA CAA CAC AGC AGG CAG GG 3’

DPE1 F 5’ ACA TCC TCG GTG ATT ACG CC 3’
DPE1 R 5’ GGA AGA TGG AAG GCT GGA CC 3’

Supplementary Table 2. Extractable enzyme activities measured *in vitro* for leaf 6 of both wild type and r*PHS1* (line 35C) plants sampled at the end of the photoperiod. All data are shown as mean of 4 independent biological replicates ± standard error of the mean and where * indicates significant difference ($p < 0.05$) between wild type and *rPHS1*. The enzyme activities are calculated as rate of production of glucose or glucose-1-P (as indicated in brackets under each enzyme) and are compared with estimated rates of nocturnal starch degradation where the units are glucose equivalents.

	Activity (nmol min^{-1} g^{-1} fwt)	
ENZYME	WT	*rPHS1* (line 35C)
Starch phosphorylase (glucose-1-P)	171 ± 22	20 ± 2 *
β-amylase (glucose)	6100 ± 520	6205 ± 439
Chloroplastic Disproportionating enzyme (glucose)	1400 ± 98	1205 ± 87
Cytosolic Disproportionating enzyme (glucose)	30 ± 2	34 ± 2
Maltase (glucose)	22 ± 4	22 ± 2
Nocturnal starch degradation (glucose equivalents)	35 ± 4 (averaged over 12 h)	17 ± 2 * (averaged over 12 h)
